# Gonorrhea in Women

**Published:** 1897-12

**Authors:** J. Henry Dowd

**Affiliations:** Buffalo, N. Y.


### GONORRHEA IN WOMEN.

By J. HENRY DOWD, M. D., Buffalo, N. Y.

OF THE more serious afflictions that are ravaging the ranks of womanhood today gonorrhea must be placed first. It must be placed before phthisis, for while life lasts the latter is bearable, but the sufferings of one whose pelvic organs are invaded by the dread germ gonococcus cannot be described better than to say she lives a life of torture every hour, from the beginning to the close of each year. After life is considered not worth living and years have been spent in painful meditation, possibly over past wrongs, possibly not knowing the cause of their sufferings, the gynecologist is sought, who at once suggests an operation for the removal of tube or ovary, or perchance to clear the pelvis of everything but the bladder, rectum and vagina. Is she now well? No. You have changed her pain, but from physical to mental; she is neither male nor female. These thoughts constantly confront her and the mental suffering at times is great.

It is not to the last division, those that are infected by husbands who perchance years before had a gonorrhea and thought

1. Read before the section on gynecology, Buffalo Academy of Medicine, October 26, 1897.

themselves well, but infected their wives with a low grade of inflammation; it is to the class where the disease is acquired acutely to which I especially desire to call attention. I will make a broad statement, but I wish to go on record as affirming that *not one woman in thirty knows when she has gonorrhea.* Lest you consider this an irrational statement let me explain.

Nature is just, just in the sense that she prevents the male from sexual indulgence while the disease is acute and when the pus is flowing from him in a stream loaded with germs; but it is, on the other hand, when chordee and discharge have subsided that he attempts what to him is pleasure but to the receiver untold suffering and worse than death. At this stage there is nothing left at the entrance, but the débris in the urethra is carried up even into the cervix uteri; thus in not 5 per cent. of cases is the urethra or vulva involved. There is no pain on urination, no swollen lips and no difficulty in walking. Unless those parts of the anatomy are involved, the woman has no symptoms that invite attention of the medical man to a possible infection, for where a woman comes complaining simply of an excess of leucorrheal discharge, as she will term it, unless a careful microscopical examination is made she will leave your office not knowing she has a specific inflammation, which, unless heroic treatment is used, rapidly extends upward, involving the tubes, ovaries and peritoneum.

Before giving the symptoms it may not be amiss to classify the different forms, that is, the different localities involved, each of which must be studied separately, treated differently and will give different symptoms. By all means the most common is cervicitis or endocervicitis. With this form we may well add endometritis, as unless the case is seen early and vigorous measures used the inflammation by continuity will extend rapidly from the cervix upward. Metritis, salpingitis, oöphoritis and peritonitis are very likely to follow.

*Vagina.*—When this is the seat of inflammation the urethra generally partakes therein, although it is by no means uncommon to find one or the other separately involved. The glands, as a general thing, take up the germs and it is from these, containing pus after the visible inflammation has been cured, that one becomes infected and another does not, although both may have been with the same woman the same night. I shall now speak of the symptomatology as urethritis, vulvitis, cervicitis or endocervicitis. The trained eye can in a large percentage of cases diagnosticate gonorrheal inflammation; not that it differs in color materially from other inflammations, but there is an angry look which when once photographed on the mind it is hard to forget. Do not let me create the impression that the microscope is not necessary; on the contrary it is an absolute necessity. I might say that in all discharges from the vagina, except possibly in virgins, two or three slides should be made and examined carefully with a 1-12 oil immersion lens.

*Vulvitis.*—Whether the urethra be involved or not there is always pain on urination. It is especially marked if it is involved. The mucous membrane is intensely red and swollen, and walking is painful, due to friction of the labiæ. If the discharge is not absorbed, but comes in contact with the skin, it will become excoriated and at times pruritus is very distressing. Separation or movement of the lips causes much pain. The small periurethral glands and those of Bartholini can at times be seen swollen and are very noticeable. Should the urethra be involved, with the finger in the vagina, stroke forward and pus will be seen to exude from the meatus. The pus in this form will be more profuse than any form, except general vaginitis. If of a golden color mixed with green this should especially call attention to the fact that you are probably dealing with something other than a simple inflammation.

*Vaginitis.*—Where the vagina is entirely involved the discharge is very profuse and of the color described in vulvitis. A temperature ranging from 100 to 104° F. is often present. A feeling of fulness with heat will be complained of, but unless the vulva is involved there is only a frequency of urination with possibly a small amount of pain as the bladder closes to expel the last few drops; there is no pain or burning, as when the urine must flow over an inflamed membrane. The inflammation, as a rule, does not involve the whole membrane, but occurs in patches the size of a nickel, intensely swollen and angry-looking. The introduction of a speculum is very painful, and even the nozzle of a syringe causes much suffering. When it is necessary to use a speculum the Ferguson is the one advisable. In withdrawing this instrument the whole mucous tract can be seen much more plainly than with the bivalve.

*Cervicitis, Endocervicitis.*—This is the form with which many a poor woman suffers, and not knowing her condition or that anything is radically wrong, goes on from month to month with the few inconveniences I shall mention until an attack of pelvic peritonitis or severe ovarian pain drives her to the physician, who after weeks of local treatment with no result, turns her over to the gynecologist. I think I am safe in saying that 90 per cent. of women who receive the gonococcus receive it at the cervix. It is in this condition they know not what they have until some careful investigator finds the germ. With a little inquiry the urine of the husband or lover will be found to contain any quantity of shreds and drops of pus, which, if examined, will be found to contain these specific cocci. The symptoms are so trivial these women do not early seek medical aid. Of course the doctor cannot be blamed if the patient does not consult him, but he is responsible if she does come and he does not use all possible means for a correct diagnosis, including the microscope.

Who, then, is at fault? Surely the cause of this specific inflammation can be traced to some one, and I most positively state it is the physician or surgeon who treated the man, discharging him as cured simply because the discharge had stopped. The discharge will be increased, increased from the normal, which all women have more or less, but not to any extent as in the cases where vulvitis or vaginitis exists. Blood may mingle therein, in fact blood cells may be found with the microscope in nearly every case.

Unless complications have set in, no pain will manifest itself. The speculum will almost decide the case, for always there will be found a cervix deeply eroded, bright red in color and having the most angry look imaginable. The vault will be filled with pus and exuding from the cervix a plug of bright yellow tinged with green. If the inflammation has involved the uterine cavity the discharge will be more profuse and possibly a feeling of fulness will be experienced by the patient.

It is not my intention to go into a description of the conditions after the inflammation has become chronic or passed out of the uterine cavity, but a few words touching this point may be of value. Generally a history of menses, extending a day or two or possibly three or four over the usual time, and that the flow is stronger and more profuse. Dysmenorrhea, which formerly was not present, is now much in evidence, and unless the trouble has extended beyond the uterus it commences with the first flow of blood. Sterility is a common complication. The discharge will be more marked after the menstrual flow has ceased.

When inflammation has passed into the tubes, ovaries and peritoneum, general symptoms will manifest themselves and to such a degree that medical aid will be sought at once. To stop the advance of gonorrheal inflammation the treatment must be vigorous, even heroic in the extreme. The fact must be borne in mind that a road leads directly from the labia majora to the peritoneal cavity. When this road once becomes inhabited with specific germs their course seems to be rapidly upward, thence to the right or left, possibly some passing both ways, distributing pioneers en route until this cavity is reached, where, blocked from further progress, they expend their force by causing inflammation of every thing in reach.

How different the female from the male sex as to treatment and complications. In the latter there is no direct opening into the peritoneum, but strong germicides cannot be used on account of the smallness of the tube, as their use would cause atresia or stricture. On the other hand, in the female, strong caustics can be used freely with no fear of resulting cicatrices. Of internal treatment very little can be said. Hygienic measures should receive careful attention. In all varieties it is necessary to keep the bowels open freely. A calomel purge, followed by a saline, should be given early and repeated every few days if thought necessary. When the vulva or urethra is involved the urine should be made bland ; should pruritus develop, a lotion of carbolic acid, 3 to 5 per cent., will usually relieve it. The diet at first should be light and easily assimilable. Later, when the inflammation has taken on a chronic form, good nutritious food is called for. A word of warning which is probably more important than all others, is the care of the hands and napkins used by such patients. They must be told of the danger should pus reach the eye, and also of the danger others undergo should they use their towels. The napkins should either be destroyed, soaked in bichloride solution, 1 to 1000, or boiled for half an hour. The hands should be thoroughly washed after each handling of the affected parts. Baths every day will be found very beneficial, and I would advise not a sitz but a general wetting of the whole body. I think it safe to say that bichloride, silver and the curette will be all that is necessary in the way of local measures, but they must be used with vigor.

With vulvitis or vaginitis, rest in bed for a few days is necessary. Where vulvitis exists alone, a thorough cleansing with bichloride, 1-4000, after which the whole mucous surface should be brushed over with a solution of silver 20 per cent., the lips separated with cotton, outside of which should be placed a pad of absorbent cotton, so that all discharge can be picked up at once. Douches of this strength of bichloride must be used three times a day and on every third day silver reapplied if necessary, the strength of which can be increased to 50 per cent. should occasion require it. When the urethra becomes involved, owing to its shortness, the inflammation rapidly reaches the bladder. After urination pass an endoscopic tube as far as the sphincter visice, but not into the bladder, and with a swab touch the mucous membrane throughout with a 10 per cent. solution of silver. By the aid of the electric light the inflammation can be watched and every third or fourth day silver used as high as 20 per cent.

*Vaginitis.*—In this condition instrumentation and local remedies, other than those used with the douche, will have to be postponed for a few days, because where the trouble is general the swelling and tenderness is great. This will especially be found if the speculum is used. The patient should be sent to bed and all hygienic and dietetic measures carried out as heretofore mentioned. Every third hour a bichloride douche, 1-5000, should be used, about half a gallon of the fluid being employed at each sitting. After a continuation of this for about three days it will be found that a speculum can be introduced without much pain, a Ferguson being the one advisable. As the mucous membrane comes into view at the end of the tube all spots of inflammation can be seen and touched ; in fact, the whole vaginal membrane can be swabbed with a 20 to 50 per cent. solution of silver. Douches should be continued morning and evening, and on the third day silver reapplied, as with the endoscopic tube in the urethra the inflammation can be watched and all areas of redness thoroughly treated until rendered normal.

Even in this day of vast research, when by the aid of the X-ray a bullet can be located in the abdominal cavity, diagnosis of the kidney involved in Bright's disease made out, or any other inflammation thereof, and we can also see the lesion in the urethra, yet we are unable to say where an inflammation ends, *i. e.*, to what area it has extended along the membrane ; therefore as a routine measure, though one I have never seen advised, where a gonorrheal inflammation has involved any portion of the female anatomy above the hymen, the uterus and cervix should be curetted.

The great and important question now arises when can curettement be done without danger of these angry cocci being taken up, floated through the system and giving rise to inflammatory conditions in the joints, endo- or pericardium and other remote tissues

On the other hand, to be of any value in preventing extension into the tubes, ovaries and other tissues, it must be done before the inflammation reaches these structures. In the male, where we can study the discharge, the extending inflammation and the appearance of the cocci, we find, I believe it is not a disputed fact, that after a certain time they enter a state of lethargy. Inflammation, then, does not extend as rapidly as heretofore unless aroused by an additional irritant or stimulant. The discharge also becomes thicker but less in quantity. Now is the time to clear them out together with all involved membrane. About the twelfth to fifteenth day from the time of infection, the condition described above will be found. All antiseptic precautions must be carefully carried out. With the woman in the dorsal position the small dilators should be used first and then one of the heavier instruments, the cervix being stretched at least three-fourths to one inch. This is absolutely necessary in order to squeeze out the contents of the cervical glands which are usually the seat or fortress of the germs we are striving to remove. The uterus should now be thoroughly curetted, together with the cervix, care being taken to clean out the fundus and openings into the Fallopian tubes. After thorough cleansing with bichloride, 1-5000, the whole cavity and that of the cervix should be thoroughly touched with a solution of silver 40 to 50 per cent. and packed, not very tightly, with iodoform gauze, which can be left in for forty-eight hours. Usually about the fourth day separation of the slough commences, during which time hot douches should be employed at least three times a day. If a careful microscopical examination is made, the discharge will be found to consist of pus cells, mucus and a few cocci, but not gonococci. A course of local treatment by iodine and ichthyol or the galvanic current, followed by local applications, will in the course of a few weeks greatly lessen, if not almost stop, this muco-pus discharge. During this treatment the bowels should move daily and tonics of iron, strychine and hydrastis be given. Sometimes it will be found necessary to repeat the curetting, applying the silver as before. The complications of gonorrhea in the female should be treated the same as though occurring in the male.

206 FRANKLIN STREET.

